# Exploring the potential duty of care in clinical genomics under UK law

**DOI:** 10.1177/0968533217721966

**Published:** 2017-08-14

**Authors:** Colin Mitchell, Corrette Ploem, Victoria Chico, Elizabeth Ormondroyd, Alison Hall, Susan Wallace, Michael Fay, Deirdre Goodwin, Jessica Bell, Simon Phillips, Jenny C. Taylor, Raoul Hennekam, Jane Kaye

**Affiliations:** University of Oxford, UK; University of Amsterdam, the Netherlands; University of Sheffield, UK; University of Oxford, UK; PHG Foundation, UK; University of Leicester, UK; Keele University, UK; 7BR Chambers, UK; University of Oxford, UK; Halebury Ltd., UK; Oxford NIHR Biomedical Research Centre, UK; Wellcome Trust Centre for Human Genetics, Oxford, UK; University of Amsterdam, the Netherlands; University of Oxford, UK

**Keywords:** Clinical genomics, duty of care, duty to warn, research duties, secondary findings

## Abstract

Genome-wide sequencing technologies are beginning to be used in projects that have both clinical diagnostic and research components. The clinical application of this technology, which generates a huge amount of information of varying diagnostic certainty, involves addressing a number of challenges to establish appropriate standards. In this article, we explore the way that UK law may respond to three of these key challenges and could establish new legal duties in relation to feedback of findings that are unrelated to the presenting condition (secondary, additional or incidental findings); duties towards genetic relatives as well as the patient and duties on the part of researchers and professionals who do not have direct contact with patients. When considering these issues, the courts will take account of European and international comparisons, developing guidance and relevant ethical, social and policy factors. The UK courts will also be strongly influenced by precedent set in case law.

## Introduction

Clinical Genetics is the branch of medicine which deals with the diagnosis, management and counselling of patients with genetic disorders. Such genetic conditions are diverse and often individually rare and include developmental disorders, dysmorphisms, inherited cancers, neurological and cardiac conditions. In providing molecular diagnoses for their patients, Clinical Geneticists have utilized sequencing technologies and array methods to provide information on pathogenic mutations in the DNA of their patients which may explain their genetic condition.^1^ Historically, sequencing methods only allowed single genes to be ‘read’ or sequenced for mutations at one time. The introduction of ‘next-generation sequencing technologies’ (NGS) enabled panels of genes to be tested simultaneously, thus significantly increasing the chances of providing patients with a molecular diagnosis for their condition.^2^ Most recently, innovations in genome sequencing technology have transformed the prospect of providing patients with genetic diseases with a molecular diagnosis.^3^ Instead of relying on knowledge of a causative gene, these methods enable the entire genome of a patient to be screened in a non-hypothesis-driven manner.^4^ While the process of genome sequencing generates very significant challenges in analysing the gigabytes of sequence data and identifying the pathogenic variant for a given disease among the thousands of naturally occurring benign variants,^5^ it has been demonstrated to increase the diagnostic yield by 20–30% over existing methods.^6^

The potential benefits of an accurate genetic diagnosis are wide ranging: they can direct appropriate medical evaluation and interventions, which can be made earlier, saving care, treatment and other costs through the patient’s lifetime. They can also direct appropriate social and educational care, provide a tool for identifying other family members at risk of disease development and provide information on which to base reproductive and other lifestyle choices.

Many large-scale research, clinical and hybrid genome sequencing initiatives are now underway, including the Genomics England 100,000 Genomes Project in the United Kingdom.^7^ This project will sequence the genomes of patients with a rare disease, some family members and patients with cancer, and aims to provide a diagnosis where there was not one before.^8^ This is one of the largest clinical sequencing projects in the world and it aims to create a new genomic medicine service for the National Health Service (NHS).

The application of genome sequencing in clinical care raises challenges that are being identified and debated around the world.^9^ Three of these – the problem of secondary findings, the extent of duties to genetic relatives and potential duties owed by researchers and non-clinical professionals – are a particular challenge for the development of new legal standards in the United Kingdom. This article aims to identify the legal duties that may arise for healthcare professionals (HCPs) and researchers in this rapidly evolving area of clinical practise. Two of the authors of this article have recently published more specific articles on aspects of the duty of care in genetic medicine in this journal. Chico focuses on the duty of clinicians to warn their patients’ families of elevated genetic risk and presents an argument for the imposition of a duty of care based on a developmental approach to ‘wrong’ and ‘harm’ in negligence.^10^ Fay also presents an argument in favour of a duty to disclose to relatives being created using the current law.^11^ This article has a broader scope and explores a range of potential novel duties, including duties to relatives that may arise in clinical genomics.

### Challenges for clinical practice

Clinical Genetics is a medical speciality concerned with assessing the probability of a condition having a genetic basis, providing a clinical and molecular diagnosis for patients, directing evaluation and management of genetic conditions appropriate to diagnosis and provision of counselling and information to support decision-making for patients and families. Other clinical specialities increasingly provide genetic services, often in tandem with Clinical Genetics as part of clinical care or research. Clinical Genetics is unusual in that it is concerned with the management of a family rather than one individual, a detailed multi-generation family history is collected and stored routinely and communication of risk to relatives is integral.^12^ Genetic testing is frequently offered, with the aim of providing a molecular diagnosis, identifying at risk family members and risk of recurrence. Single gene and multigene panels are being supplemented with ‘whole exome sequencing’ (WES) or ‘whole genome sequencing’ (WGS) particularly when the condition is genetically heterogeneous or when no causative mutation is detected in gene panel testing (at present in clinical care WES is used, but it is expected that WGS will be the norm in just a few years’ time). Assessments of predicted pathogenicity of genetic variants are made by qualified clinical scientists, who typically issue a report including those variants considered highly likely or likely to be pathogenic, as well as those of uncertain pathogenicity (variant of uncertain significance). Confirmed genetic variants are then reported by an accredited laboratory to the referring HCP. In reaching this assessment, all available molecular, family segregation and population data are evaluated. However, due to the complexity and (often very) limited nature of these data, different laboratories sometimes reach different conclusions about the pathogenicity of a given variant. Variation in developing practice may be problematic in determining the standard of care that could be considered reasonable in the law.

#### Secondary findings

A major issue for emerging practice is that of genomic variants that are considered secondary to the presenting health condition. These have often been called ‘incidental findings’ and characterized as unanticipated variants of potential clinical significance that are unrelated to the disease that prompted the sequencing.^13^ However, because secondary results are sometimes specifically sought, or should be expected as part of WES/WGS,^14^ other terms have been suggested, such as secondary findings^15^ or additional findings.^16^ Management of secondary findings is challenging: they are likely to be unexpected, and, in an individual who appears not to have any symptoms of the secondary condition, the significance is uncertain. This raises questions about the standard of care that may be considered reasonable in law.^17^ Should such variants be reported? If so, how and under what circumstances? What medical follow-up is indicated? According to which guidelines? Should uncertain results be updated if they later become considered pathogenic and by whom? Recommendations for practice will guide the UK courts and legal authorities in their considerations of the duty of care,^18^ and there are developing recommendations on best practice in this context.

One approach, recommended by the American College of Medical Genetics and Genomics (ACMG), is to screen a specific list of genes in individuals of all ages undergoing clinical exome and genome sequencing. The ACMG proposed a list of 56 genes that are associated with later onset conditions and/or have a long asymptomatic phase, including inherited cancer predisposition and inherited heart conditions. The ACMG suggests that genomic variants of ‘known’ (or in some cases ‘expected’) pathogenicity be routinely reported back to physicians and their patients unless the patient opts out of this analysis.^19^ It is estimated that such variants might occur in around 1–7% of individuals.^20^ Some experts are cautious of this approach.^21^ Perhaps the greatest concerns are that the evidence that a pathogenic variant will give rise to a disease in an individual who has no other signs of the disease is much less clear, and that this could ultimately give rise to reporting of false positives with the potential for harm due to anxiety, unwarranted screening and risk management interventions.^22^ The ACMG acknowledges this problem of correctly predicting ‘penetrance’ (the likelihood of the disease manifesting), and their approach is to curate the list of genes in light of any new evidence revising estimates of penetrance – potentially removing genes from the list.^23^

By contrast, in order to minimize the challenges presented by secondary findings, the European Society of Human Genetics (ESHG) recommends an approach to WES/WGS that reduces the potential for generating incidental findings. They recommend an initial targeted analysis of genes known to be associated with the presenting condition in and continuing to unrestricted analysis of whole exome/genome data only if a causative variant is not identified in the targeted analysis.^24^ The ESHG recommends that a protocol should be developed that sets out the approach that will be taken to incidental findings and that, in principle, a HCP should report an unsolicited finding if it is indicative of serious health problems, unless the patient indicated beforehand that he did not want to know.^25^ The PHG Foundation similarly recommends an approach to limit the generation of incidental findings but, if found, recommends that the decision to feedback is guided by the consent process and ‘informed by clinical judgment.’^26^

In current practice, decision-making pathways for such results may involve the use of a multi-disciplinary team (MDT) or advisory board to guide the evaluation and reporting of secondary findings.^27^ Although promoting a thorough analysis and consensus in decision-making, this is a resource and labour-intensive approach to result interpretation that may not be feasible at scale.^28^ Approaches that are informed by professional judgment also have the potential to create significant differences in relation to the standard of care, which could pose difficult questions for the standard required by law. The generation of large amounts of genetic information and the uncertainty involved also complicates the process of obtaining a patient’s informed consent to sequencing. Informed consent is of central importance in clinical genomics, not only because it is legally required to legitimize the taking of samples and the secondary use of genomic data^29^ but also because adequate information and communication is required to help patients understand the potential consequences of genome or exome sequencing and make autonomous choices about the information they may receive.^30^ Some suggestions have been made to enhance the level of autonomous choice that patients have over the return of secondary findings. One proposed approach is the separation of results into a number of potential categories, termed ‘bins’ with decisions about feedback made by the patient for each category of potential result.^31^ Another possible approach is a mixed version, whereby certain categories of results (e.g. actionable and child-onset conditions) are automatically returned as part of the standard of care.^32^ However, ensuring informed consent in genomics is challenging, particularly if information is provided at a single clinic visit prior to testing.^33^ There is no clear position in current practice. In some projects, patients may be asked whether they wish for a search for secondary or incidental findings. Alternatively, patients may simply consent to sequencing on the basis that they *may* be informed of certain results if the clinicians consider them important – for example, if they are both serious and actionable. This is current practice in some countries such as the Netherlands. If patients do not want to be informed of these findings, the sequencing will not be performed using WES or WGS techniques.

#### Blurring of research and clinical activity

Approaches to secondary findings are complicated by the fact that the technology is moving from the research domain into a clinical realm and currently diagnostic exome/genome sequencing is used in a way that combines research and clinical care.^34^ Projects such as the 100,000 Genomes Project, which recruits patients and relatives within the NHS for diagnostic purposes (there is a mandatory feedback policy for primary or pertinent findings), also have research aims. This is arguably part of an established tradition for clinical genetics, where existing clinical diagnostic tests have sometimes been supplemented with research-based tests.^35^ However, research and clinical care traditionally have different goals. The goal of clinical care is to provide a benefit to the patient, whereas research is designed to produce generalizable knowledge to benefit society, and participants do not necessarily receive results from research. These differences have been reflected in the legal framework, and, while HCPs clearly owe legal duties to their patients, researchers have been less likely to owe duties to participants, particularly those with whom they have little or no contact. When research and care become blurred, for example, when patients with undiagnosed conditions turn to exome or genome sequencing for answers, ‘ascertaining when clinical care has morphed into research (or vice versa) can be quite difficult’^36^ and there may be an increased danger of ‘therapeutic misconception’ (a misguided assumption that the researcher is acting exclusively in the best interest of the individual patient).^37^ To ensure that participants understand the differences between their clinical care and genomic research and to ensure that researchers are clear in their obligations and not unnecessarily diverted from producing important generalizable knowledge, it is important that research and clinical pathways are distinguished as clearly as possible.^38^ As we consider the section ‘Is a duty of care owed by researchers, scientists and non-clinical professionals?’, although the courts are likely to take into account the different resources and aims of clinical care and research, where there is close interaction between care and research, there is the real possibility that genomics researchers will be found to owe a legal duty to disclose findings to participants.

#### The rights and interests of genetic relatives

A third challenge for genetics and genomics in medicine, whether using individual gene tests or genome-wide approaches, is the incorporation of familial interests as well as those of the patient or participant. Patient confidentiality is a cornerstone of healthcare and, although many patients may be happy to disclose genetic information to relatives, the extent of HCPs or researchers’ legal obligations towards family members when the patient refuses disclosure is unclear.^39^ Currently, the default position within existing guidance is for HCPs to treat information as confidential to the individual patients and disclosure without consent as a rare exception.^40^ However, there have been powerful ethical arguments that a different approach to information sharing and confidentiality should be taken in genetic medicine. Parker and Lucassen argue that, because genetic information is ‘spontaneous mutations aside, essentially and unavoidably familial in nature’,^41^ it should be treated as held on a ‘joint account’ with other family members.^42^ Doing so would shift the default position from patient confidentiality to one where the information from clinical genetic testing is routinely shared with family members unless the patient is at risk of serious harm. Gilbar adopts a relational approach to autonomy which emphasizes that the ‘patient develops his or her autonomy by engaging with those around him or her’,^43^ to argue that the effect on the dynamics of a family should be one of the criteria used by professionals in deciding whether to share information with relatives. This argument draws on empirical evidence that patients and professionals do take a largely familial approach to genetic testing,^44^ an approach that has been reflected in practise and guidelines.^45^ However, there are challenges to more family-centric approaches.^46^ Liao argues that, considering the possibility of spontaneous mutations and incomplete penetrance of some variants, risk to relatives may be lower than at first sight and only in a few cases is the significance of the information sufficient to override confidentiality.^47^ It is also questioned whether guidance to disclose information to relatives takes sufficient account of the possibility of the relatives having an interest in not knowing (a right not to know).^48^ Because it is impossible to make a free choice about not knowing genetic information, Laurie argues that this interest is best protected by respecting the relative’s privacy, which should only be invaded if good cause is shown.^49^ The effect of this is not to prevent disclosure but to require that HCPs consider how an individual might react and the strength of the clinical considerations justifying disclosure. While the ethical debate includes a range of nuanced approaches to the interests of genetic relatives, the law in the United Kingdom has, until recently, denied claims made by family members for a failure to warn of genetic risk. As we discuss below, this is a new area of legal challenge and there are reasons to believe that some claims by genetic relatives may succeed. This issue arises in all forms of genetic testing, but genomic technologies potentially increase the amount of information that may be valuable to a relative.^50^ In this article, we explore the potential legal duties that may develop in the United Kingdom in response to these key challenges for clinical practice and analyse how these duties may be determined in the courts.

## Establishing novel legal duties for clinical genomics

Our analysis is focused on the potential existence and nature of a *legal* duty of care in UK law. There is no legislation on the duties involved in genome sequencing in the United Kingdom, and, in the absence of this, any new legal duties on the part of professionals in clinical genomics will be established within the common law of negligence. Some direction on potential duties may be derived from international human rights documents, such as the treaties of the Council of Europe. The Convention on Human Rights and Biomedicine^51^ and its Additional Protocols set out that an individual is entitled to know any information collected about his or her health,^52^ including information collected during research^53^ and any information ‘collected about his or her health derived’ from a genetic test for health purposes.^54^ The distinction between research and clinical genetic testing under these instruments is that results of research testing should only be offered if they are of relevance to health; this is not likely to be established for many genetic results without follow-up investigation and validation. The Convention also suggests that diagnosis should not extend beyond the complaints of the patient – limiting the use of wider tests where narrower tests are available.^55^

However, because the United Kingdom is not a signatory to the Oviedo Convention, it does not imply the existence of a legal duty of care within the established rules and principles of the law of negligence. In negligence, a duty of care may be owed to take reasonable care not to injure others who it could be reasonably foreseen would be harmed by action or inaction.^56^ The most common test for establishing a novel duty of care in negligence was set out by the House of Lords in *Caparo Industries plc. v. Dickman*.^57^ This test requires that three criteria must be fulfilled before a duty of care is imposed: foreseeability of the harm, proximity and that it is ‘just, fair and reasonable’ to impose a duty. This third requirement is a broad one that can include consideration of policy factors. When a duty of care is established, the legal standard of care will also be determined (often as part of an overlapping analysis), along with determinations of whether or not there has been a breach of that duty.

The challenges for clinical genomics highlighted above give rise to three key legal questions within the law of negligence. First, will there be legal duties to provide secondary findings when identified? Second, are duties owed to relatives as well as patients? Third, are duties owed by researchers and other professionals who do not normally have direct interactions with patients?

### Is there a duty to feed back secondary findings?

One of the key ethical issues for genome sequencing is whether there is a duty to investigate and feed back secondary findings. Determining a legal duty will be a question of whether some parts of practice should be subject to a legal duty of care and, if a duty of care is found to exist, the appropriate standard of care. As outlined above, currently a range of different approaches are taken concerning secondary findings (results that are not pertinent to the condition under immediate investigation). The ACMG recommends active investigation of a number of selected genes while other organizations, such as the ESHG, prefer a targeted approach to testing that limits the discovery of incidental findings in genes.

#### A ‘reasonable’ approach to secondary findings

There is as yet no established legal duty to look for or return secondary findings in the United Kingdom, so a claim relating to such findings would need to satisfy the court that it would be ‘fair, just and reasonable’ to impose a duty. In practise, this is a wide-ranging assessment that can include consideration of current policies, standards and the potential burden for clinicians and scientists in identifying, interpreting and validating secondary findings. To actively look for findings could be a more onerous obligation than to validate and return findings that are discovered unintentionally during analysis – true incidental findings. As we discuss further below, when WES or WGS is carried out as a research endeavour, a duty to either look for or report secondary findings is even less likely to be considered fair, just and reasonable. However, the boundary between clinical care and practise is not well defined for genomics and, as these techniques begin to be used as clinical tests, and the relationship between the parties becomes more clearly that of clinical care team and patient, it is more likely that a duty to feed back certain kinds of secondary findings will develop. In practise, the existence and nature of that duty will be determined by answering both whether a duty would be reasonable and what the appropriate standard of care is in deciding which secondary findings should be validated and returned to patients.

In terms of the standard of care, in English law, the standard expected of a skilled professional is generally that of a reasonably competent member of that specialism or profession; if a doctor reaches the standard of a responsible body of medical opinion, he is not negligent. In medical negligence, the assessment of what is reasonable is largely guided by the *Bolam* test^58^: action must be in accordance with a practise accepted as proper by a responsible body of medical opinion, as long as this practise could not be rejected as incapable of standing up to rational analysis (the qualification made in the *Bolitho* case).^59^ In such a new area, the possibility for divergence among professionals makes determining an appropriate legal standard more complicated.

In the United Kingdom, the 100,000 Genomes Project has developed an intermediate approach to secondary findings – between the ACMG and ESHG positions. It has developed a list of genes in which a very limited number of known, pathogenic mutations of high clinical relevance are investigated if the participant consents. This list is more limited than that developed by the ACMG but also includes optional carrier status for a small number of recessive conditions where both parents participate and X-linked carrier status in women.^60^ Participants are offered screening of genes in these categories, if appropriate, during the process of seeking informed consent, and their choices are recorded on the consent form. In accordance with Good Clinical Practice, a copy is stored in the participant’s hospital notes and a copy is sent with their sample for central storage. As described above, feedback of incidental findings may involve an MDT or advisory board of experts to help evaluate results, based on expected pathogenicity and other factors.^61^ This process could therefore fulfil the classic *Bolam* and *Bolitho* tests, as long as the development of the gene lists and decisions about results are well founded. Even when a field is developing and approaches are not uniform (as with the differences between ACMG and ESHG recommendations), it is likely that courts will treat well-founded guidance as evidence of a responsible body of medical opinion.^62^ However, this does not necessarily end the debate on the standard of care required in dealing with secondary findings. As we will see below, the courts in England and Wales have taken a new direction in some cases concerned with disclosure of medical risks and have applied a standard that reflects more closely what a patient might reasonably want to know.^63^ This case law could complicate the assessment of appropriate standards if it is found to apply to the disclosure of genomic information to patients.

#### Patient-centric disclosure?

The ACMG and 100,000 Genome Project secondary findings gene lists use clinical ‘actionability’ as the main criterion for inclusion. However, there is limited evidence concerning the relative importance of this or other factors for stakeholders, including participants. For example, knowledge of possession of certain genetic variants might be considered important by individuals, even if not clinically actionable or even relevant to healthcare, as part of a fulfilment of a right to self-determination and autonomy. Some results that are not considered clinically actionable would be important to some patients in making future decisions about their health and reproduction. The courts in the United Kingdom have required a more patient-centric approach to information disclosure in some aspects of medical practise, including determining what information a patient should be provided with as part of the consent process. This patient-centric approach could influence the courts in deciding which information should be given to patients during clinical genome sequencing.

Historically, in the context of consent and disclosure of risk, the courts endorsed the reasonable professional’s (*Bolam*) standard for the disclosure of risk and rejected a standard based on what the reasonable patient would want to know; suggesting that it is generally for the doctors to decide what risks should be communicated.^64^ However, more recently, the Supreme Court (SC) handed down unanimous judgment in the case of *Montgomery v. Lanarkshire Health Board* which sets a new standard on disclosure of risk.^65^ This was a claim brought by a patient, Mrs Montgomery, who gave birth to a child who sustained severe brain damage as a result of complications in labour caused by shoulder dystocia. She claimed that doctors had negligently failed to warn her of the risk of shoulder dystocia and that, had she been warned, she would have asked for a surgical delivery. It was agreed at trial that the general risk of shoulder dystocia in diabetic mothers – such as Mrs Montgomery – is around 9–10%. However, the responsible doctor said that it was not her practise to discuss this risk because she felt that the risk of ‘grave problems’ resulting from shoulder dystocia was very small but that despite this, most women would instead choose caesarean section if so warned. The SC unanimously found in favour of Mrs Montgomery^66^ and set out a new test for the disclosure of risk in medical law, clarifying several decades of uncertain case law in the process. The SC emphasized that the *Bolam* test of ‘conduct supported by a responsible body of medical opinion’ is inappropriate in the context of risk disclosure and consent. The court held that it would be wrong to regard the previous leading case of *Sidaway* ^67^ as an unqualified endorsement of the application of the *Bolam* test to the giving of advice about treatment. Instead, they considered that Lord Woolf MR’s assessment in *Pearce* ^68^ was correct in deciding that a ‘significant risk which would affect the judgment of the reasonable patient’ should be disclosed.^69^ Following *Montgomery*, the test to determine whether a risk is ‘material’ and should be disclosed is now whether:

[A] reasonable person in the patient’s position would be likely to attach significance to the risk or if the doctor should reasonably be aware that this particular patient would be likely to attach significance to it.^70^

The SC drew a distinction between the *Bolam* standard of a reasonable body of medical opinion that should still apply to decisions about which investigatory or treatment options should be considered and the provision of information about risks, which does not require special *medical* skill:

The former role is an exercise of professional skill and judgement: what risks of injury are involved in an operation, for example, is a matter falling within the expertise of members of the medical profession. But it is a *non sequitur* to conclude that the question whether a risk of injury, or the availability of an alternative form of treatment, ought to be discussed with the patient is also a matter of purely professional judgment. The doctor’s advisory role cannot be regarded as solely an exercise of medical skill without leaving out of account the patient’s entitlement to decide on the risks.^71^

This decision was influenced by the importance of the value of self-determination under Article 8 of the European Convention on Human Rights as considered by the courts since the *Sidaway* case^72^ and by the Convention on Human Rights and Biomedicine.^73^ In her concurring judgment, Lady Hale also emphasized that the interests which the law of negligence now protects include ‘a person’s interest in their own physical and psychiatric integrity, an important feature of which is their autonomy’ and agreed with Jonathan Herring that the issue is whether enough information is provided to give ‘due protection to the patient’s right of autonomy’.^74^

This test has already been applied to genetic information. In *Mrs A v. East Kent University NHS Foundation Trust*, it was claimed that a material risk of a chromosomal abnormality should have been disclosed to Mrs A during her pregnancy.^75^ This was a very rare chromosomal abnormality with only one other potentially similar case known worldwide. The judge found that the risk of chromosomal abnormality from the evidence at the time was too slight (well below 1%) to require further investigation. Applying *Montgomery*, the judge also found that there was no reason to discuss such a risk because ‘a reasonable patient, in the position of Mrs A, would have attached no significance to risks at this background level. Further…I do not find Mrs A would have attached significance to these levels of risk.’^76^ However, it is currently uncertain quite how *Montgomery* would apply in the context of clinical genomics and whether this standard could apply to the disclosure of results and information about potential diagnoses rather than the risks of intervention and consent.

In terms of consent and the risks of intervention, *Montgomery* now suggests that consent to genome sequencing requires careful consideration of which risks are ‘material’ and potentially significant to an individual patient. Whether this applies to non-physical ‘risks’ is unclear; Could this extend to a material risk of psychological harm due to uncertainty of results, for example? In order to be ‘material’ this would need to be a risk of inflicting a recognized psychiatric disorder as a result of such uncertainty. It cannot be concluded that the ‘reasonable patient’ standard will be transplanted to apply in the context of disclosure of findings, but it is clear that *Montgomery* sets a more subjective standard that considers the position of the specific patient. This judgment and its partial approval for the principle of autonomy as a basis for information standards may signal a more expansive approach in this area and could support a duty to disclose wider findings than HCPs have decided to return if a patient may find them important for self-determination (for example, for reproductive choices). Alternatively, *Montgomery* also makes clear that there is still a narrow ‘therapeutic exception’ to disclosure of risk where it is reasonable to withhold information if it would be seriously detrimental to a patient’s health (although the judgment suggests that this would be a rare occurrence).^77^ The existence of this ‘therapeutic privilege’ holds out the prospect of an exception to disclosure, it could be argued, to prevent significant psychological harm from disclosure of wider and less certain WGS results.

The key question in terms of feedback of secondary findings is what test would be applied to determine what is reasonable? A standard based on a reasonable body of medical opinion or a standard that seeks to provide protection to a patient’s right to self-determination and autonomy? This matter is further complicated in the case of the developing child in pregnancy cases or for minors, whose autonomy might instead provide a reason not to disclose adult-onset conditions.

### Are duties owed to relatives in Clinical Genomics?

The question of whether there ought to be a duty of care to inform family members of information that might affect their health or life choices has always been central to Clinical Genetics practice, and the introduction of WES/WGS may increase the likelihood of such information coming to light. HCPs do not generally owe duties to those who fall outside their care according to English Law,^78^ and there is a significant weight of case law against duties to third parties in negligence,^79^ the general rule being that HCPs do not owe a duty to non-patients and third parties.^80^ However, there are factors that suggest genetic relatives may be successful in claims based on a failure to warn. The increasing importance of this issue is demonstrated by two recent cases.

#### ABC v. St George’s Healthcare NHS Trust

In *ABC*, the claimant was the daughter of a man diagnosed with Huntington’s disease. She claimed that a duty of care was owed to warn her of the possibility that she had inherited the condition and could pass it on to her children, against the wishes of her father who had refused to disclose this piece of confidential information.^81^ The claimant was not party to a doctor–patient relationship and this potential duty of care to a ‘third party’ was recognized by the judge as ‘entirely novel’.^82^ On the basis of the parties’ submissions, Mr Justice Nicol concluded that there was ‘no reasonably arguable duty of care’,^83^ and the claim was struck out. This was largely on the basis that such a duty would conflict with the well-established duty of confidentiality owed by doctors to their patients and could not be seen as an incremental extension of the law of negligence.^84^ For these and other reasons, Nicol J concluded that imposing a duty of care would not be fair, just and reasonable.

There are a number of factors in favour of a duty of care that were not considered as part of Nicol J’s analysis. In this regard, reference can be made to a number of legal scholars arguing that some form of duty to relatives could be anticipated,^85^ and factors that have led to courts in other jurisdictions, for example, in States of the United States, taking a broader approach. In *Pate v. Threlkel*,^86^ the Florida SC found a duty to warn the patient that their children may have a risk of illness. This approach was approved by the Minnesota SC in *Molloy v. Meier*,^87^ which found a duty was owed to biological parents to conduct Fragile X testing of their child and to communicate the diagnosis to them, although this did not require direct communication with the parents by those conducting the test.^88^ However, the Superior Court of New Jersey Court in *Safer v. Estate of Pack* took a broader approach – drawing on infectious disease case law – to find a duty to warn a non-patient daughter directly. A key factor in reaching this decision was that the group to which a duty would potentially be owed was easily identified and that there would only be a duty to warn directly where substantial harm could be averted or minimized. The impact of *Safer* has been tempered by the New Jersey Genetic Privacy Act, which prohibits disclosure of genetic information (that could identify the patient) without consent but its reasoning remains a useful precedent in the common law.^89^ These cases were not considered by Nicol J.

Although it may not be easy to fit a duty to warn alongside the established duty of confidentiality, it is clear in English law that a breach of confidence may be justified to protect important rights and to prevent serious harm.^90^ There are indications that many patients are willing to forgo their confidentiality for relatives to be informed,^91^ and most recent professional guidance also acknowledges that genetic information may be relevant to the patient’s relatives and that patients should be informed of this.^92^ Established clinical practice is to facilitate and empower patients to communicate genetic risk to their relatives, through provision of tailored letters addressed ‘to whom it may concern’. Guidance from the General Medical Council (GMC) on confidentiality acknowledges that informing relatives against the express wishes of a patient could be justified in certain circumstances where another person is at risk of ‘serious harm’.^93^ In determining whether to disclose against a patient’s consent and in the ‘public interest’, the guidance emphasizes that only a serious risk could outweigh the patient’s and public interest in confidentiality.^94^ It is unclear precisely how a risk of serious harm would be interpreted in the context of genetic risk, but it is clearly possible that a risk of serious illness or death (e.g., where a BRCA1 or BRCA2 gene mutation is identified in a parent, conferring a serious increased risk of breast cancer if inherited by a daughter) which could be avoided or ameliorated by early preventative measures or treatment could outweigh a patient’s confidentiality. This guidance suggests that it is good practice to inform a patient about the relevance of information to their relatives and advise them to disclose risks to family. GMC guidance also suggests it may be justified to disclose directly to relatives, even against the patient’s express wishes, in some extreme circumstances. The rationale for this type of approach was set out by the Joint Committee on Medical Genetics on Consent and Confidentiality in Clinical Genetic Practice: ‘The assumption that confidentiality is always paramount is as inappropriate as the assumption that disclosure is always permissible, and the decision will need to be tailored to the individual circumstances of the case.’^95^ The SC has also made clear that it may be desirable for the law to set the standards required in the performance of professional duties.^96^

A highly persuasive aspect of the application to strike out the claim in *ABC v. St George’s Healthcare NHS Trust* was that duties of care should be extended only incrementally from some well-established legal duty, and the judge instead felt that recognizing a duty to warn genetic relatives would be a ‘giant step’ in the law’s development (referring to the judgment of Lord Toulson in *Michael v. Chief Constable of the South Wales Police*). However, this admittedly well-established point could be challenged in these specific circumstances. As Lord Toulson himself makes clear, argument by incremental analogy is only part of the analysis and it may be that an ‘earlier limitation is no longer logically or socially justifiable’ and also that ‘[o]ften there will be a mixture of policy considerations to take into account’.^97^ Significant social and justice considerations are at least arguable in the case of genetic relatives at risk of serious harm. There are also some indications that the courts are able to contemplate a duty of care to the non-patient where they are a family member who is bound up in the same circumstances, such as the care and treatment involved in pregnancy. In the case of *Anderson v. Forth Valley Health Board*, the court found a duty of care in prenatal genetic testing was owed to the father, despite it being the mother who was the patient.^98^ Moreover, Clinical Genetics practice, although recognizing the confidential nature of information from the individual, where possible, clearly acknowledges the familial nature of genetic information. By extending the legal duty of care to close genetic relatives, arguably this would be a narrow extension to a limited and identifiable group who could benefit from a duty.^99^ This type of duty could be seen as a more incremental extension of the law than was suggested by the defendants in *ABC*.

The claimant appealed against Nicol J’s decision to strike out her claim. The Court of Appeal decided to allow her appeal, agreeing that a duty of care was arguable and that such arguments should be made in full at trial.^100^ Handing down the unanimous judgment, Irwin LJ found scope for reasonable argument in most aspects of the defendants’ submissions. It was recognized that there were no direct precedents for this claim in English law but Irwin LJ did approve parallels in foreign cases, including *Safer v. Pack*, as support for an argument that the extension of a duty to relatives in clinical genetics could be a reasonable, incremental development of the law of negligence.^101^ Irwin LJ closely analysed California SC decisions in *Tarasoff v. Regents of the University of California* ^102^ and *Rowland v. Christian* which were forerunners of the favourable decision in *Safer* and where, in *Rowland*, it was recognized that

[t]he court have carved out an exception to [the rule against third party disclosure] in cases in which the defendant stands in some special relationship to either the person whose conduct needs to be controlled or in a relationship to the foreseeable victim of that conduct.^103^

We accordingly await the full trial for an answer on whether, following the Court of Appeal analysis and acknowledgement that a duty to third parties may exist, such duty of care will be found to have existed in the circumstances of *ABC*.

#### Connor Smith v. University of Leicester NHS Trust

The initial judgment in *ABC* was quickly followed by a second case, *Connor Smith* (heard when the appeal in *ABC* was pending), where the claims of non-patient relatives were also struck out.^104^ In this case, it was alleged that there had been a negligent delay in testing a patient, the claimants’ second cousin Mr Craven, for adrenomyeloneuropathy (AMN). This is a genetic disease affecting the brain. But for this negligent delay – of more than 3 years in testing for AMC – it was claimed that the condition would have been detected and family members would have also been tested. The claimants, Connor and Callum Smith, claimed they could have been diagnosed earlier with the childhood version of the condition and given treatment that might have led to a significantly better outcome for both boys. The test for AMN had originally been requested by Mr Craven’s clinician in 2003 but did not take place. It was only once the boys had been diagnosed in 2006 that the same Consultant Neurologist noticed his request had not been carried out and the test was ordered again. As in *ABC*, His Honour Judge McKenna struck out the claim, finding that it ‘would not be fair, just and reasonable on policy grounds to impose a duty of care on the defendant in respect of those who are not its patients’.^105^ He concluded that it was the settled policy of the law not to grant ‘remedies to third parties for the effects of injuries to other people and what the claimants seek in this case is to introduce an exception to that approach’.^106^ No exception had succeeded in the past and it would not be fair, just and reasonable, in his opinion, for it to succeed in this case.

Although there are similarities with *ABC* – a claim by non-patient genetic relatives that they should have been warned of genetic risk – this was not a case based on non-disclosure of available information involving a conflict with patient confidentiality. The claimants argued that this difference restricted the relevance of *ABC* ^107^ but this was rejected by the Judge, who decided that Nicol J’s decision in *ABC* set a precedent against a duty of care to non-patients in general and was not restricted to circumstances involving patient confidentiality. Unlike *ABC*, the issue of proximity between the defendant and the claimants was contested and it is clear that the distance between them was a concern for the Judge who decided that ‘to extend the duty of care to the patient’s second cousins’ would be unreasonable.^108^ He was unmoved by the argument that there was an assumption of responsibility by the NHS Trust towards the wider family or that this point should at least be argued at a full hearing.^109^ Some of the same criticisms can be made of this court decision as of Nicol J’s decision in *ABC.* There was little consideration of the familial approach of clinical genetics and the fact that extending a duty of care to genetic relatives in certain circumstances would not be a giant step for clinicians, who are already very aware of familial implications. The court also failed to consider the extension of duties in other jurisdictions to reflect this.

However, *Connor Smith* is a different case to those based on a failure to warn relatives of a genetic diagnosis. The distance between the parties (the daughter in *ABC* was known to the clinicians) was greater in a physical sense and, as opposed to *ABC*, the defendants may not have even been aware of the existence of the second cousins.^110^ In this case, the claimed duty was not to warn relatives that they were at risk of illness but – arguably a more onerous duty – to perform genetic testing in the patient, in order to provide risk information for their relatives. This is one-step forward from discovery of information which could benefit relatives. It is also a potentially onerous duty to find and contact relatives who are physically further away and perhaps not well known to either the patient or clinicians. And, although the lack of conflict with patient confidentiality in this case removes a major element of the argument against a duty that was so persuasive in the initial application to strike out in *ABC*, carrying out testing, as opposed to disclosure of results, is not an aspect of practice where the guidance in the United Kingdom explicitly recommends the consideration of relatives’ interests. Indeed the Court of Appeal in *ABC* acknowledged this aspect when commenting upon the important distinction of the unpredictable nature of risks to potential victims and the possibility of unnecessary warnings being given in a general clinical environment and clinical genetics, where testing has already been carried out: ‘the obligation will usually arise from a specific quantifiable risk. Indeed it is one of the clinical functions of the geneticist to calculate the risk’.^111^

Although the UK courts may be more likely to recognize a duty to advise the patient of risk to relatives, these recent challenges demonstrate there is a case to be argued that a duty of care exists – at least in some circumstances where the proximity between the parties is sufficiently close – to warn relatives directly of genetic risks. As genome-wide testing begins to be used in clinical care, more results will be discovered that might have clinical significance for relatives and the legal duties involved are likely to be tested further.

This and the previous section have analysed the potential legal response to aspects of developing practice in clinical genomics, the next section will consider which professionals may owe a duty to patients, participants or their relatives.

### Is a duty of care owed by researchers, scientists and non-clinical professionals?

A key legal question given the current interdisciplinary nature of genome sequencing is whether, and in what circumstances, the professionals involved in clinical WES/WGS owe a legal duty of care to the patient? In addition to patient-facing HCPs, a range of other professionals such as clinical lab scientists, bioinformaticians and researchers may be involved in the generation, analysis and interpretation of genome sequence data. As a starting point, the clinical care team owe a duty of care to their patient. As set out in the *Caparo* formulation, a duty of care will only exist if there is a relationship of proximity between the person who owes the duty (such as a doctor) and the subject of the duty (the patient). The concept of proximity involves the notion of nearness or closeness and includes physical proximity (in the sense of space and time), circumstantial proximity, causal proximity and assumption of responsibility.^112^ It is well established in law that doctors owe a duty of care to patients because of the ‘close direct relationship’ between them.^113^ For a duty of care to be found on the part of professionals who are not part of the clinical care team, a claimant would need to establish proximity and persuade the court that such a duty would be ‘fair, just and reasonable’. The analysis of this will depend on the nature of the duty alleged – for example, to feed back certain findings – but there are some factors that will be influential in most circumstances.

It was established in *The Creutzfeldt-Jakob Disease Litigation* that researchers may owe a duty of care to research participants ‘akin to that of doctor and patient, one of close proximity.’^114^ However, this duty was found in a clinical trial which then became a part of medical practice. Perhaps because this was a therapeutic programme and very close to clinical care, a duty of care was established without considering proximity between researchers and participants thoroughly. Following this, it has been suggested that the taking on of a clinical role by the professional or the close relationship between research and therapy could be persuasive in finding a duty of care.^115^ It could be argued that those professionals who can be said to play a ‘therapeutic role’ in clinical genomics might be subject to a duty of care to patients. But this invites a potentially difficult question – What constitutes ‘therapeutic’? Determining this may be particularly difficult where non-clinically qualified individuals contribute to provision of clinical information, as may be the case with research results from ‘hybrid’ genomics projects, or where the ‘researcher’ is a clinician carrying out research alongside their clinical work.

Previous judicial consideration concerning the difficulty of determining a boundary between clinical activities and research undertaken by clinicians took place in *Walker Smith v. GMC*, a review of a decision by the Fitness to Practise Panel of the Royal College of Physicians (RCP).^116^ Drawing on clinical guidance at the time, Mitting J found that the clinician’s intentions were relevant to determining whether an activity was research or medical care. However, it was also clear that experimental medical care need not constitute research as long as its goal was the care of the patient and that there was a ‘reasonable chance of success’.^117^ That test is subjective and in the judge’s view

When the person undertaking the activity has two purposes or when different people participating in the same series of activities have different purposes, it may be very difficult to say into which category the activities fall. This difficulty is particularly likely to arise in activities undertaken by an academic clinician and/or in a teaching hospital with a research department.^118^

This indicates some factors that could be important in assessing whether or not a duty of care would be ‘fair, just and reasonable’ in the law of negligence; it is possible that a clinician would be considered to be carrying out clinical activity (and therefore should meet clinical standards of care) where a goal of the project is also the care of the patient. This could be the case, despite the labelling of sequencing as ‘research’ or obtaining of research ethics approval. In other jurisdictions, including other common law jurisdictions such as Canada, it has also been established that researchers owe a duty of care to research participants.^119^ If the decisions made by these wider professionals shape the knowledge, care and information provided to the patient, there may be reasons under that heading which could persuade a court that such a duty would be ‘fair, just and reasonable’. To understand how the courts might approach this, further analysis is required of the roles of all the professionals involved in genomics; their influence on patient care and the expectations that are developed in relevant professional or ethical guidance.

A second potential source of liability for these wider professionals is a duty of care towards the clinical care team for the reasonableness, accuracy and competence of their work. In a sense, this could be seen as analogous with the duty of care of professional advisors towards other professionals – a duty of care one step removed from the patient. Researchers could be liable to patients in a way analogous to surveyors^120^ or expert third parties (e.g., testing laboratories^121^) in that the advice/service is provided to the clinic, not the patient, but the patient is reliant on appropriate information being passed to their clinical care team. In these circumstances, the patient could be argued as reasonably within the contemplation of the researcher, suggesting that a duty could be fair, just and reasonable. As practice develops, particularly in the investigation and analysis of wider findings from genomics, the potential existence and scope of such duties would benefit from further analysis.

## Conclusions on the duty of care in clinical NGS

This article identifies novel duties that may arise in UK law in the context of genome and exome sequencing in the clinic. First, a duty may be found to return certain findings that do not pertain to the presenting health condition, either on a case-by-case basis if they are discovered ‘incidentally’ or as part of a standard investigation of some ‘additional’ findings if evidence accumulating in the field suggests that this is reasonable. Although there is currently no consensus on return of findings from genomics, as genome sequencing becomes a clinical (rather than solely research) test, a duty of care could well be established to provide significant incidental or additional results. From a legal perspective, this view raises to important questions of the legal standard of care in clinical sequencing. This issue may need separation into two parts. First, a standard of professional skill and competence will apply to decisions about what should be investigated, which results are analysed fully and whether results should be re-evaluated in the future. This standard is one of practice in accordance with a responsible body of medical opinion (the *Bolam* standard), and in many cases, this may be straightforward. A range of approaches to which findings should automatically be investigated or excluded is likely to be reasonable given the current state of the art and guidance on management of incidental findings. Second, a different standard may apply to the practice of consent and return of results following the case law that emphasizes the respect for a patient’s autonomy and right to self-determination. Following *Montgomery*, it is clear that all the ‘material risks’ that a reasonable patient (or a particular patient) would attach significance to should be disclosed as part of the consent process. This might extend to disclosure when discussing a general course of action, such as continuing with a pregnancy. Whether material risks also include less direct harms, such as psychological harm due to uncertainty in WES/WGS results, are unclear. The tenor of this case law suggests increasing respect for self-determination and autonomy, and this may influence the courts’ assessment of the duty of care as it applies to the return of genomic results to patients as well. This would be in line with the European human rights framework and Article 10 of the Biomedicine Convention, which asserts a right to information about personal health.

A second novel duty of care is also plausible, a duty of care to warn genetic relatives directly in very limited circumstances. This has been found to be at least arguable by the Court of Appeal in *ABC*, albeit highly dependent on the factual circumstances in which it is argued that such a duty exists. The latest medical guidance suggests a professional duty to consider warning relatives of a significant genetic risk of serious harm and such a duty has been found in other jurisdictions. The courts in England and Wales have been reluctant to impose liability towards those who are not technically seen as ‘patients’ of the defendant, but we argue that such an approach is insufficiently informed by contemporary practise in clinical genetics and genomics and the Court of Appeal utterances in *ABC* appear to support this argument.

Third, it is possible that a duty of care to investigate and feed back results could be found on the part of some researchers and wider professionals (non-clinicians) in genomics towards patients if there are sufficient factors of ‘proximity’ and considerations that would make such a duty ‘fair, just and reasonable’. These are more likely to be found if a researcher also has a therapeutic role or the research project aims to have a potential impact on therapeutic outcomes or management. Further work is required that analyses the precise relationships and roles of professionals in genomics against the factors considered by the courts as part of the questions of proximity and whether a duty of care is fair, just and reasonable.

As projects such as the 100,000 Genomes Project proceed in the United Kingdom, further analysis of the legal issues outlined in this article is required. However, an attempt to establish the parameters of a duty of care from one holistic doctrine is difficult. In practice, the development of duties and their limits within the courts is not easy to predict and will depend on the individual circumstances of the case and the factors that a particular court considers most important at the time. For clinicians and professionals involved in hybrid projects and clinical genomics, a more practical approach might be to set out a framework for decision-making involving a set of principles and factors to take into account in different circumstances. For clinicians or researchers, decisions involve consideration of whether a duty of care exists and to whom, the scope of the duty, whether it is appropriately discharged and ultimately the consequences of not doing so. The legal issues outlined in this article should be considered by those developing decision-making guidance and frameworks used by HCPs and researchers.

## Notes

1. H.L. Rehm, ‘Disease-Targeted Sequencing: A Cornerstone in the Clinic’, *Nature Reviews Genetics* 14 (2013), p. 295.

2. S. Goodwin, J.D. McPherson and W. Richard McCombie, ‘Coming of Age: Ten Years of Next-Generation Sequencing Technologies’, *Nature Reviews Genetics* 17 (2016), p. 333.

3. L.G. Biesecker and R.C. Green, ‘Diagnostic Clinical Genome and Exome Sequencing’, *New England Journal of Medicine* 370 (2014), p. 2418.

4. J.C. Taylor et al., ‘Factors Influencing Success of Clinical Genome Sequencing across a Broad Spectrum of Disorders’, *Nature Genetics* 47 (2015), p. 717.

5. C.F. Wright et al., ‘Policy Challenges of Clinical Genome Sequencing’, *British Medical Journal* 347 (2013), p. f6845. DOI: https://doi.org/10.1136/bmj.f6845.

6. Taylor et al., ‘Factors Influencing Success’, 2015; C. Gilissen et al., ‘Genome Sequencing Identifies Major Causes of Severe Intellectual Disability’, *Nature* 511 (2014), p. 344.

7. Genomics England is a wholly owned company of the UK Department of Health.

8. Available at: www.genomicsengland.co.uk/the-100000-genomes-project (accessed 10 February 2017).

9. A comprehensive deliberation of the ethical, legal and social issues for clinical genomics in the United Kingdom, based on a series of workshop discussions with HCPs and other experts has been carried out by the PHG Foundation in their Realising Genomics project: A. Hall, T. Finnegan and C. Alberg, *Realising Genomics in Clinical Practice* (Cambridge: PHG Foundation, 2014).

10. V. Chico, ‘Non-Disclosure of Genetic Risks The Case for Developing Legal Wrongs’, *Medical Law International* 1–2 (2016), pp. 3–26.

11. M. Fay, ‘Negligence, Genetics and Families a Duty to Disclose Actionable Risks’, *Medical Law International* 3–4 (2016), pp. 115–142.

12. S. Dheensa et al., ‘Health-Care Professionals’ Responsibility to Patients’ Relatives in Genetic Medicine: A Systematic Review and Synthesis of Empirical Research’, *Genetics in Medicine* 18 (2016), pp. 290–301; R. Gilbar, ‘Communicating Genetic Information in the Family: The Familial Relationship as the Forgotten Factor’, *Journal of Medical Ethics* 33 (2007), p. 390; M. Parker and A.M. Lucassen, ‘Genetic Information: A Joint Account?’, *British Medical Journal* 329 (2004), p. 165.

13. N. Tan et al., ‘Is “Incidental Finding” the Best Term?: A Study of Patients’ Preferences’, *Genetics in Medicine* 19 (2017), pp. 176–181.

14. J.Y. Hehir-Kwa et al., ‘Towards a European Consensus for Reporting Incidental Findings During Clinical NGS Testing’, *European Journal of Human Genetics* 23 (2015), p. 1601.

15. S.S. Kalia et al., ‘Recommendations for Reporting of Secondary Findings in Clinical Exome and Genome Sequencing, 2016 Update (ACMG SF v2.0): A Policy Statement of the American College of Medical Genetics and Genomics’, *Genetics in Medicine* 19 (2017), p. 249.

16. L. Eckstein, J.R. Garrett and B.E. Berkman, ‘A Framework for Analyzing the Ethics of Disclosing Genetic Research Findings’, *Journal of Law, Medicine & Ethics* 42 (2014), p. 190.

17. A.L. McGuire et al., ‘Can I Be Sued for That? Liability Risk and the Disclosure of Clinically Significant Genetic Research Findings’, *Genome Research* 24 (2014), p. 719; M.C. Ploem, ‘Handling Unsolicited Findings in Clinical Care: A Legal Perspective’, *European Journal of Health Law* 21 (2014), p. 489; E.W. Clayton et al., ‘Managing Incidental Genomic Findings: Legal Obligations of Clinicians’, *Genetics in Medicine* 15 (2013), p. 624.

18. The Supreme Court has recently demonstrated the importance of authoritative guidelines in setting a legal standard of care in *Montgomery v. Lanarkshire Health Board* [2015] UKSC 11; [2015] 2 WLR 768.

19. The initial recommendation in 2013 was that some actionable results should be returned to patients without an opt-out. ACMG Board of Directors, ‘ACMG Policy Statement: Updated Recommendations Regarding Analysis and Reporting of Secondary Findings in Clinical Genome-Scale Sequencing’, *Genetics in Medicine* 17 (2015), p. 68.

20. S. Schuol et al., ‘So Rare We Need to Hunt for Them: Reframing the Ethical Debate on Incidental Findings’*, Genome Medicine* 7 (2015), p. 83.

21. C.F. Wright et al., ‘Policy Challenges’, 2013; C.G. van El et al., ‘Whole-Genome Sequencing in Health Care’, *European Journal of Human Genetics* 21 (2013), p. S1.

22. Hehir-Kwa et al., ‘Towards a European Consensus’, p. 1602.

23. Kalia et al., ‘Recommendations for Reporting of Secondary’, p. 253.

24. C.G. Van El et al, ‘Whole-Genome Sequencing in Health Care: Recommendations of the European Society of Human Genetics’, *European Journal of Human Genetics* 21 (2013), pp. 580–584. DOI: 10.1038/ejhg.2013.46.

25. Mandatory feedback of additional findings is, however, normal practise in some parts of Europe: T. Rigter et al., ‘Reflecting on Earlier Experiences with Unsolicited Findings: Points to Consider for Next-Generation Sequencing and Informed Consent in Diagnostics’, *Human Mutation* 34 (2013), p. 1322.

26. Hall, Finnegan and Alberg, *Realising Genomics*, Recommendation 9.

27. E. Ormondroyd et al., ‘Insights from Early Experience of a Rare Disease Genomic Medicine Multidisciplinary Team: A Qualitative Study’, *European Journal of Human Genetics* 25 (2017), pp. 680–686. DOI: 10.1038/ejhg.2017.37.

28. T. Rigter et al., ‘Informed Consent for Exome Sequencing in Diagnostics: Exploring First Experiences and Views of Professionals and Patients’, *Clinical Genetics* 85 (2014), p. 417.

29. Data Protection Act 1998, sch. 3, para. 1.

30. V. Chico, ‘Known Unknowns and Unknown Unknowns: The Potential and the Limits of Autonomy in Disclosure of Genetic Risk’, *Professional Negligence* 28 (2012), p. 162.

31. J.S. Berg, M.J. Khoury and J.P. Evans, ‘Deploying Whole Genome Sequencing in Clinical Practice and Public Health: Meeting the Challenge One Bin at a Time’, *Genetics in Medicine* 13 (2011), p. 499.

32. H.J. Jacob et al., ‘Genomics in Clinical Practice: Lessons from the Front Lines’, *Science Translational Medicine* 5 (2013), p. 194cm5.

33. P.S. Appelbaum et al., ‘Models of Consent to Return of Incidental Findings in Genomic Research’, *Hastings Center Report* 44 (2014), p. 22.

34. S. Dheensa et al., ‘Management of Incidental Findings in Clinical Genomic Sequencing Studies’, *eLS* (2016), pp. 1–7. DOI: 10.1002/9780470015902.a0025838.

35. Hall, Finnegan and Alberg, *Realising Genomics*, p. 13.

36. B.E. Berkman, S. Chandros Hull and L. Eckstein, ‘The Unintended Implications of Blurring the Line Between Research and Clinical Care in a Genomic Age’, *Personalized Medicine* 11 (2014), p. 285, p. 286.

37. Hall, Finnegan and Alberg, *Realising Genomics*, p. 77.

38. G.P. Jarvik et al., ‘Return of Genomic Results to Research Participants: The Floor, the Ceiling, and the Choices in Between,’ *American Journal of Human Genetics* 94 (2014), p. 818; Berkman, Chandros Hull and Eckstein, ‘The Unintended Implications’, 2014, p. 293.

39. Dheensa et al., ‘Health-Care Professionals’ Responsibility’, pp. 290–301.

40. S. Dheensa, A. Fenwick and A. Lucassen, ‘“Is This Knowledge Mine and Nobody Else’s? I Don’t Feel That.” Patient Views about Consent, Confidentiality and Information-Sharing in Genetic Medicine’, *Journal of Medical Ethics* 42 (2016), pp. 174–179.

41. Parker and Lucassen, ‘Genetic Information’, 2004.

42. Op. cit., p. 165.

43. Gilbar, ‘Communicating Genetic Information in the Family’, 2007.

44. R. Gilbar, ‘The Passive Patient and Disclosure of Genetic Information: Can English Tort Law Protect the Relatives’ Right to Know?’, *International Journal of Law, Policy and the Family* 30 (2016), p. 79, p. 81.

45. S. Dheensa, A. Fenwick and A. Lucassen, ‘Approaching Confidentiality at a Familial Level in Genomic Medicine: A Focus Group Study with Healthcare Professionals’, *British Medical Journal Open* 7 (2017), p. 9. DOI: 10.1136/bmjopen-2016-012443.

46. D. Bell and B. Bennett, ‘Genetic Secrets and the Family’, *Medical Law Review* 9 (2001), p. 130.

47. S.M. Liao, ‘Is There a Duty to Share Genetic Information?’, *Journal of Medical Ethics* 35 (2009), p. 306.

48. G.T. Laurie, ‘Recognizing the Right Not to Know: Conceptual, Professional, and Legal Implications’, *Journal of Law, Medicine & Ethics* 42 (2014), p. 53.

49. Op. cit.

50. A.M. Lucassen and R.S. Houlston, ‘The Challenges of Genome Analysis in the Health Care Setting’, *Genes* 5 (2014), p. 576.

51. Convention for the Protection of Human Rights and Dignity of the Human Being with regard to the Application of Biology and Medicine: Convention on Human Rights and Biomedicine (opened for signature 4 March 1997, entered into force 1 December 1999) CETS No. 164.

52. Convention on Human Rights and Biomedicine, Article 10 (2).

53. Additional Protocol to the Convention on Human Rights and Biomedicine Concerning Biomedical Research (2005) Article 26.

54. Additional Protocol to the Convention on Human Rights and Biomedicine, concerning Genetic Testing for Health Purposes (2008) Article 16 (2).

55. Ploem, ‘Handling Unsolicited Findings in Clinical Care’, p. 499.

56.
*Donoghue v. Stevenson* [1932] AC 562.

57.
*Caparo Industries plc v. Dickman* [1990] 2 AC 605.

58.
*Bolam v. Friern Hospital Management Committee* [1957] 1 WLR 582.

59.
*Bolitho v. City and Hackney Health Authority* [1997] UKHL 46; [1998] AC 232; [1997] 4 All ER 771.

60. Genomics England, 2015, ‘The 100,000 Genomes Project Protocol’, 16 May. Available at: www.genomicsengland.co.uk/download/100000-genomes-project-protocol-2015-17/, at 6.1 (accessed 21 June 2015).

61. This approach is taken in Oxford where genome sequencing is being carried out by the NIHR Biomedical Research Centre; a collaboration between Oxford University Hospitals NHS Foundation Trust and the University of Oxford (www.oxfordbrc.nihr.ac.uk). It is also the approach of genomic medicine centres in the Netherlands: Rigter et al., ‘Reflecting on Earlier Experiences with Unsolicited Findings’, p. 1324.

62. A. Samanta et al., ‘The Role of Clinical Guidelines in Medical Negligence Litigation: A Shift from the Bolam Standard?’, *Medical Law Review* 321(14) (2006), p. 321, at p. 344.

63.
*Montgomery v. Lanarkshire Health Board* [2015] UKSC 11.

64.
*Sidaway v. Board of Governors of the Bethlem Royal Hospital* [1985] AC 871; [1985] 2 WLR 480.

65.
*Montgomery v. Lanarkshire Health Board* [2015] UKSC 11.

66. There has been significant debate of this decision. For example, Jonathan and Elsa Montgomery criticize the court’s assessment of the evidence in the case and of the reality of medical decision-making in line with best practise in these circumstances. Farell and Brazier are more supportive of the decision: J. Montgomery and E. Montgomery, ‘Montgomery on Informed Consent: An Inexpert Decision?’, *Journal of Medical Ethics* 42 (2016), p. 89; A.M. Farell and M. Brazier, ‘Not So New Directions in the Law of Consent? Examining *Montgomery v. Lanarkshire Health Board*’, *Journal of Medical Ethics* 42(2) (2015), pp. 85–88.

67.
*Sidaway v. Board of Governors of the Bethlem Royal Hospital* [1985] AC 871; [1985] 2 WLR 480.

68.
*Pearce v. United Bristol Healthcare NHS Trust* [1999] PIQR P 53.

69. Although in *Pearce* Lord Woolf MR appeared to define ‘significant’ from the perspective of the doctor.

70.
*Montgomery v. Lanarkshire Health Board* [2015] UKSC 11 [87].

71. Op. cit., para. 83.

72. Op. cit., para. 80.

73. Convention for the Protection of Human Rights and Dignity of the Human Being with regard to the Application of Biology and Medicine: Convention on Human Rights and Biomedicine (opened for signature 4 March 1997, entered into force 1 December 1999) CETS No. 164.

74. J. Herring, *Medical Law and Ethics*. 4th ed. (Oxford: OUP, 2012), p. 170.

75.
*Mrs A v. East Kent Hospitals University NHS Foundation Trust* [2015] EWHC 1038 (QB); [2015] Med LR 262.

76. Op. cit., para. 89.

77.
*Montgomery v. Lanarkshire Health Board* [2015] UKSC 11 [85], [88] & [91].

78.
*Powell v. Boladz* (1997) 39 BMLR 35; [1998] Lloyd’s Rep Med 116, CA.

79. R. Mulheron, *Medical Negligence: Non-Patient and Third Party Claims* (Ashgate Publishing, Ltd, 2010), p. 8; Perhaps the most recent powerful authority against a duty to third parties is the House of Lords decision in *Mitchell & Anor v. Glasgow City Council* [2009] UKHL 11; [2009] 2 WLR 481, Farnham.

80. Mulheron, *Medical Negligence*, p. 18.

81.
*ABC v. St George’s Healthcare NHS Trust & Ors* [2015] EWHC 1394 (QB); [2015] Med LR 307.

82. Op. cit., para. 23.

83. Op. cit., para. 31.

84. This conclusion has been contested. For example see: R. Gilbar and C. Foster, ‘Doctors’ Liability to the Patient’s Relatives in Genetic Medicine; *ABC v. St George’s Healthcare NHS Trust* [2015] EWHC 1394 (QB)’, *Medical Law Review* 24 (1) 2016, pp. 112–123. DOI: 10.1093/medlaw/fwv037; C. Mitchell et al., ‘A Duty To Warn Relatives in Clinical Genetics: Arguably ‘Fair just and reasonable’ in English Law?’, *Tottel’s Journal of Professional Negligence* 32 (2016), pp. 120–136.

85. M. Fay, ‘Informing the Family: A Geneticist’s Duty of Care to Disclose Genetic Risks to Relatives of the Proband’, *Professional Negligence* 27 (2011), p. 97; L.J. Deftos, ‘Genomic Torts: The Law of the Future – The Duty of Physicians to Disclose the Presence of a Genetic Disease to the Relatives of Their Patients with the Disease’, *University of San Francisco Law Review* 32 (1997), p. 138; S.L. Keeling, ‘Duty to Warn of Genetic Harm in Breach of Patient Confidentiality’*, Journal of Law and Medicine* 12 (2004), p. 235; Mulheron, *Medical Negligence*, 2010.; V. Chico, *Genomic Negligence: An Interest in Autonomy as the Basis for Novel Negligence Claims Generated by Genetic Technology* (Abingdon: Taylor & Francis, 2011).

86.
*Pate v. Threlkel* (1995) 661 So 2d 278.

87.
*Molloy v. Meier*, 679 N.W.2d 711(Minn. 2004).

88.
*Safer v. Estate of Pack* (1996) 677 2d 1188 (Appellate Div).

89. Fay, ‘Negligence, Genetics and Families’, p. 11. However, there may be some caution in the relevance of US case law for UK legal development. As J. Moorland suggests in *The CJD Litigation*; ‘The culture of American personal injury litigation is very different from ours and thus public policy considerations’ (1997) 41 BMLR 157, [161]-[162].

90. This is discussed further by Graeme Laurie in G.T. Laurie, *Genetic Privacy: A Challenge to Medico-Legal Norms* (Cambridge: Cambridge University Press, 2007), p. 219.

91. T.J. Heaton and V. Chico, ‘Attitudes Towards the Sharing of Genetic Information with At-Risk Relatives: Results of a Quantitative Survey’, *Human Genetics* 135 (2015), p. 109.

92. A. Lucassen and A. Hall, ‘Consent and Confidentiality in Clinical Genetic Practice: Guidance on Genetic Testing and Sharing Genetic Information’, *Clinical Medicine* 12 (2012), p. 5.

93. General Medical Council, 2009, ‘Confidentiality Guidance: Genetic and Other Shared Information’, 12 October. Available at: www.gmc-uk.org/guidance/ethical_guidance/confidentiality_67_69_genetic_and_other_shared_information.asp (accessed 20 November 2014), paras 67–69.

94. Op. cit., para. 55.

95. Royal College of Physicians, Royal College of Pathologists and British Society for Human Genetics, 2011, ‘Report of the Joint Committee on Medical Genetics; Consent and Confidentiality in Clinical Genetic Practice: guidance on genetic testing and sharing genetic information,’ September 2011. Available at: www.bsgm.org.uk/media/678746/consent_and_confidentiality_2011.pdf (accessed 21 June 2015).

96. Per. Lord Kerr and Lord Reed; *Montgomery v. Lanarkshire Health Board* [2015] UKSC 11 [93].

97.
*Michael & Ors v. The Chief Constable of South Wales Police & Anor* [2015] UKSC 2; [2015] AC 1732, [102].

98.
*Anderson v. Forth Valley Health Board* [1998] S.L.T. 588

99. Dheensa et al., ‘Health-Care Professionals’ Responsibility’, pp. 290–301.

100.
*ABC v. St George’s Healthcare NHS Trust & Others* [2017] EWCA Civ 336.

101. Op. cit., paras 46–63.

102.
*Tarasoff v. Regents of the University of California* (1976) 551 P.2d 334.

103.
*Rowland v. Christian* (1968) 69 Cal.2d 108.

104.
*Smith & Anor v. University of Leicester NHS Trust* [2016] EWHC 817 (QB).

105. Op. cit., para. 30.

106. Op. cit., para. 29.

107. Op. cit., para. 24.

108. Op. cit., para. 29.

109. Op. cit., paras 26–27.

110. Although the Consultant was aware of them by 2006; Op. cit., para. 8.

111.
*ABC v. St George’s Healthcare NHS Trust & Others* [2017] EWCA Civ 336, 56.

112.
*Sutherland Shire Council v. Heyman* (1985) 60 A.L.R. 1.

113.
*Barnett v. Chelsea and Kensington Hospital Management Committee* [1968] 1 All ER 1068.

114. The Creutzfeldt-Jakob Disease Litigation (1997) QB 41 BMLR 157, 164.

115. J. Kaye et al., *Ethical, Legal and Social Issues Arising from the Use of GWAS in Medical Research*, March 2009, Available at: https://wellcome.ac.uk/sites/default/files/wtx058032.pdf (accessed 21 June 2015).

116.
*Walker-Smith v. General Medical Council* [2012] EWHC 503 (Admin).

117. Op. cit., para. 16.

118. Op. cit.

119.
*Halushka v. University of Saskatchewan* [1965] 52 W.W.R. 608 (Sask. C.A.).

120.
*Smith v. Eric S Bush (A Firm)* [1990] UKHL 1; [1989] 2 WLR 790.

121.
*Farraj & Anor v. King’s Healthcare NHS Trust* (KCH) & Ors [2009] EWCA Civ 1203.
